# The CXCR4 and adhesion molecule expression of CD34+ hematopoietic cells mobilized by “on-demand” addition of plerixafor to granulocyte–colony-stimulating factor

**DOI:** 10.1111/trf.12632

**Published:** 2014-03-28

**Authors:** Tamara Girbl, Verena Lunzer, Richard Greil, Konrad Namberger, Tanja Nicole Hartmann

**Affiliations:** Laboratory for Immunological and Molecular Cancer Research, Third Medical Department with Hematology, Medical Oncology, Hemostaseology, Rheumatology and Infectiology, Paracelsus Medical UniversitySalzburg, Austria

## Abstract

**Background:**

Granulocyte–colony-stimulating factor (G-CSF) is routinely used for mobilization of hematopoietic stem and progenitor cells preceding autologous transplantation after high-dose chemotherapy in hematologic malignancies. However, due to high mobilization failure rates, alternative mobilization strategies are required.

**Study Design and Methods:**

Patients who poorly mobilized CD34+ hematopoietic cells (HCs) with G-CSF additionally received the CXCR4 antagonist plerixafor. The phenotype of CD34+ HCs collected after this plerixafor-induced “rescue” mobilization, in regard to adhesion molecule and CD133, CD34, and CD38 expression in comparison to CD34+ HCs collected after traditional G-CSF administration in good mobilizers, was analyzed flow cytometrically. To confirm previous studies in our patient cohort, the efficiency of mobilization and subsequent engraftment after this “on-demand” plerixafor mobilization were analyzed.

**Results:**

Pronounced mobilization occurred after plerixafor administration in poor mobilizers, resulting in similar CD34+ cell yields as obtained by G-CSF in good mobilizers, whereby plerixafor increased the content of primitive CD133+/CD34+/CD38– cells. The surface expression profiles of the marrow homing and retention receptors CXCR4, VLA-4, LFA-1, and CD44 on mobilized CD34+ cells and hematopoietic recovery after transplantation were similar in patients receiving G-CSF plus plerixafor or G-CSF. Unexpectedly, the expression levels of respective adhesion receptors were not related to mobilization efficiency or engraftment.

**Conclusion:**

The results show that CD34+ HCs collected by plerixafor-induced rescue mobilization are qualitatively equivalent to CD34+ HCs collected after traditional G-CSF mobilization in good mobilizers, in regard to their adhesive phenotype and engraftment potential. Thereby, plerixafor facilitates the treatment of poor mobilizers with autologous HC transplantation after high-dose chemotherapy.

Autologous hematopoietic cell (HC) transplantation is widely applied to reconstitute hematopoiesis after high-dose chemotherapy in patients with hematologic malignancies including multiple myeloma (MM), non-Hodgkin's lymphoma (NHL), and Hodgkin's lymphoma.^1^,^2^ Mobilized peripheral blood (PB) CD34+ hematopoietic stem and progenitor cells (HSPCs) are easy to collect, have a high engraftment potential, and are used as preferred source for transplantation. To mobilize HSPCs from the marrow (BM) into the PB, their adhesive interactions with accessory stromal cells, osteoclasts,^3^ osteoblasts, and extracellular matrix components in the BM need to be overcome. The chemokine CXCL12 and the α_4_β_1_ (VLA-4, CD49d/CD29) integrin ligand VCAM-1 are abundantly expressed by BM stromal cells and act as key mediators of HSPC retention in the BM.^4^ In line, disruption of CXCL12–CXCR4 and VCAM-1–VLA-4 interactions results in release of HSPCs into the PB.^5-7^ Additionally, the α_L_β_2_ integrin LFA-1 (CD11a/CD18) and the adhesion receptor CD44 are involved in HSPC lodgment in the BM and CD44 is also implicated in mobilization of these cells.^8-10^ Importantly, the receptors CXCR4, VLA-4, LFA-1, and CD44 also mediate BM homing of HSPCs,^9,11-14^ wherefore their expression might influence the migration of these cells into the BM and engraftment after autologous transplantation.

Currently, the granulocyte–colony-stimulating factor (G-CSF) is used as standard mobilizing agent, alone or in combination with chemotherapy. G-CSF causes severe alterations of the BM microenvironment including osteoblast apoptosis,^15^ depletion of CD68+/CD169+ BM macrophages,^16^,^17^ inhibition of osteogenic mesenchymal stem cell differentiation,^18^ and bone formation.^16^,^19^ Collectively, these alterations cause a down regulation of molecules important for HSPC maintenance and retention including CXCL12, VCAM-1, SCF, and angiopoietin-1^16-20^ in endosteal and vascular niches, which eventually facilitates HSPC mobilization. G-CSF has been suggested to further promote HSPC mobilization by inducing the expression of the proteases matrix metalloproteinase-9, cathepsin G, and neutrophil elastase, which together with mediators of the complement cascade^21-23^ and thrombolytic pathway^24^ cleave and inactivate the retention factors CXCL12, VCAM-1, and c-Kit.^25-29^ However, in 10% to 30% of all patients G-CSF fails to efficiently mobilize CD34+ HSPCs and does not support the collection of at least 2 × 10^6^ CD34+ cells/kg body weight in one collection attempt, which are minimally required for transplantation and successful engraftment after high-dose chemotherapy.^30-33^ These patients require alternative mobilization regimens. Plerixafor (AMD3100), a bicyclam antagonist of CXCR4, which reversibly blocks CXCL12 binding and chemotactic signaling,^34^,^35^ has recently been identified as potent mobilizing agent. Broxmeyer and colleagues^7^ first showed that plerixafor and G-CSF act synergistically in the mobilization of functionally competent human stem cells. Subsequent studies demonstrated that plerixafor elevates CD34+ cell harvests in comparison to G-CSF alone in healthy volunteers^36^ and NHL and MM patients.^37-39^ Therefore, plerixafor was approved in combination with G-CSF for HSPC mobilization for autologous transplantation in lymphoma and MM patients.

In our study G-CSF was used as standard mobilizing agent. Patients identified as “poor mobilizers,” based on low circulating CD34+ cell counts during G-CSF administration, additionally received plerixafor (“on demand”). As plerixafor induces HSPC mobilization by mechanisms distinct from G-CSF, it may release HSPCs of different subsets and different adhesion and migration receptor expression profiles. We evaluated HSPC subset composition based on CD133, CD34, and CD38 expression and the surface expression of CXCR4, CD49d (α-subunit of VLA-4), CD11a (α-subunit of LFA-1), and CD44 on collected CD34+ cells. Additionally, we analyzed the mobilization efficiency of plerixafor “rescue” administration in comparison to standard G-CSF application, to confirm previous investigations demonstrating plerixafor efficiency^37,39-44^ in our patient cohort. To further compare the quality of mobilized HCs, we evaluated the time to engraftment after transplantation and the impact of CXCR4 and adhesion molecule expression on time to neutrophil and platelet (PLT) engraftment.

## Materials and Methods

### Patients and HSPC collection

Under approval from the local ethics committee (No. 415-E/1177/8-2010) and after written informed consent was obtained, 37 patients with hematologic malignancies (summarized in Table 1 and detailed in Table S1, available as supporting information in the online version of this paper), who underwent HSPC mobilization at the Third Medical Department of the Paracelsus Medical University Salzburg, were included in this study. Mobilization was induced by daily subcutaneous injections of 10 μg of G-CSF (Merckle Biotec, Ulm, Germany; or Novartis, Basel, Switzerland) per kg of body weight for 3 to 4 consecutive days before leukapheresis was started. A total of 75.7% of our patients additionally received chemotherapy (Table 1), which was followed by daily administration of 10 μg/kg G-CSF for 7 to 12 days starting from the day after chemotherapy. Leukapheresis was initiated at the discretion of the attaining physician and depended on circulating CD34+ cell numbers as monitored via a flow cytometer (FC-500, Beckman Coulter, Fullerton, CA). A total of 20 × 10^6^ CD34+ cells/L PB were considered as minimum and at least 50 × 10^6^ CD34+ cells/L PB as optimum to induce leukapheresis. If the minimum cell number was not attained during G-CSF administration, patients were considered as “poor mobilizers” and received a single subcutaneous injection of 240 μg/kg plerixafor followed by induction of leukapheresis after 10 to 12 hours. Cell collection was performed in one session with a commercially available apheresis system (COBE Spectra, Terumo BCT, Lakewood, CO). The number of collected CD34+ cells was determined by flow cytometer. Patients not responding to plerixafor were not subjected to leukapheresis and therefore not included in this study.

**Table 1:** Patient characteristics

Characteristics	Mobilization regimen	
	G-CSF	G-CSF plus plerixafor
Number of patients	29	8
Age (years)	54 (18-73)	62 (30-72)
Male sex	20 (69.0)	1 (12.5)
Chemotherapy previous to G-CSF	24 (82.8)	4 (50.0)
Chemotherapy regimen		
Cyclophosphamide	15 (51.7)	3 (37.5)
R-DHAP	3 (10.3)	1 (12.5)
DHAP	4 (13.8)	0
BEACOPP	1 (3.4)	0
CHOEP	1 (3.4)	0
Diagnosis		
NHL	10 (34.5)	4 (50.0)
MM	14 (48.3)	3 (37.5)
Hodgkin's lymphoma	3 (10.3)	0
Acute lymphocytic leukemia	1 (3.4)	0
Acute myeloid leukemia	1 (3.4)	0
Scleromyxedema	0	1 (12.5)

* Data are reported as median (range) or number (%).

R-DHAP = rituximab–dexamethasone, cytarabine and cisplatin; DHAP = dexamethasone, cytarabine, and cisplatin; BEACOPP = bleomycin, etoposide, doxorubicin, cyclophosphamide, vincristine, procarbazine, and prednisone; CHOEP = cyclophosphamide, doxorubicin, vincristine, and prednisone plus etoposide.

### Flow cytometry

Fresh leukapheresis products were stained with the following monoclonal antibodies (MoAbs): anti-CXCR4–PE, anti-CD49d–PE, anti-CD11a–PE, anti-CD34–FITC (BD Biosciences, Franklin Lakes, NJ) or corresponding isotype controls. Additionally, PB mononuclear cells (PBMNCs) were isolated by density gradient centrifugation, viably frozen, and stored in liquid nitrogen until use. Thawed PBMNCs were stained with anti-CD34–FITC (BD Biosciences), CD44–FITC, anti-CD38–PE, anti-CD34–PE (Beckman Coulter), and anti-CD133–APC MoAbs (Miltenyi Biotec, Bergisch-Gladbach, Germany). The percentage of cells expressing respective receptors and mean fluorescence intensity values in relation to isotype controls (MFIRs) were analyzed with a flow cytometer and its accompanying software (Gallios and Caluza, respectively, both Beckman Coulter).

### Definition of engraftment

Neutrophil engraftment was defined as the first day after transplantation where neutrophil counts in the PB reached at least 500 × 10^6^/L. PLT engraftment was defined as first (of 3 consecutive) day(s) where PLT counts reached at least 50 × 10^9^/L PB based on guidelines of the European Group for Blood and Marrow Transplantation.^45^ In three cases (marked in Table S1), we observed lower but stable PLT counts in the absence of bleeding without further requirement of PLT transfusion at discharge from the hospital, which was defined as engraftment.

### Statistical analyses

Statistical analyses were performed with computer software (GraphPad, San Diego, CA). The Kolmogorov-Smirnov test was used to evaluate normal distribution. Normally distributed data were analyzed by t test, nonnormally distributed by Mann-Whitney test. Correlation analyses were carried out using the Pearson's test for normally distributed or Spearman test for nonnormally distributed data. Two-tailed p values of less than 0.05 were considered significant.

## Results

### G-CSF and G-CSF plus plerixafor result in the mobilization of CD34+ cells with similar CXCR4 and adhesion molecule expression profiles

**Figure 1:**
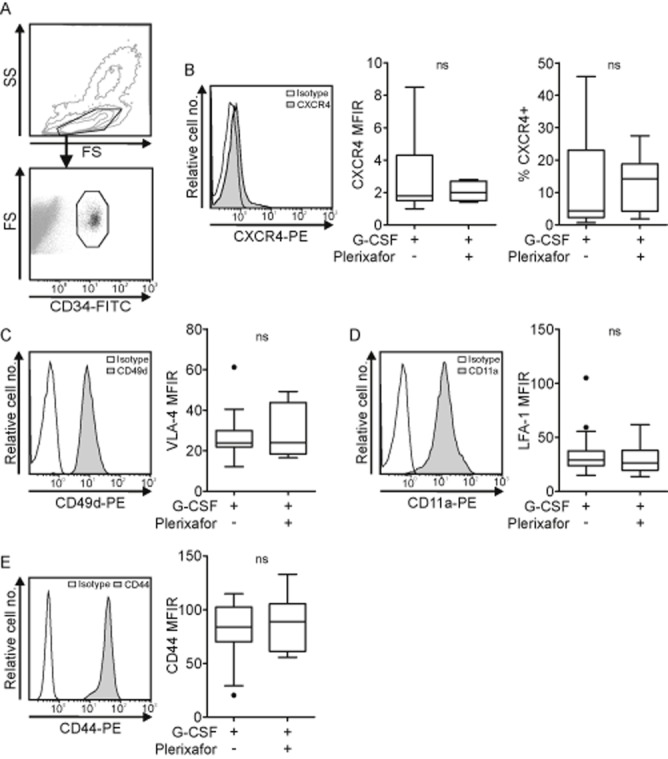
CXCR4 and adhesion molecule expression profiles in G-CSF and G-CSF plus plerixafor–mobilized HSPCs. (A) Representative density and dot plots illustrate the gating strategy for HSPCs based on forward and side scatter (FS and SS) and CD34 expression. (B) Representative fluorescence histograms (left), MFIRs of surface CXCR4 levels (middle), and percentages of CXCR4+ cells (right) of CD34+ cells collected after G-CSF– (n = 23) or G-CSF plus plerixafor–induced mobilization (n = 7). Representative fluorescence histograms of CD49d (C, left), CD11a (D, left), and CD44 (E, left) expression on CD34+ cells mobilized with G-CSF or G-CSF plus plerixafor. The corresponding MFIRs of CD49d (C, right, n_(G)_ = 24, n_(G+P)_ = 7), CD11a (D, right, n_(G)_ = 24, n_(G+P)_ = 7), and CD44 (E, right n_(G)_ = 15, n_(G+P)_ = 6) are shown in box plot format. G = G-CSF; G+P = G-CSF plus plerixafor. ns = not significant.

The surface expression of CXCR4, VLA-4, LFA-1, and CD44, which are involved in HSPC BM homing and retention, were measured on CD34+ cells in leukapheresis products collected after G-CSF plus plerixafor–induced rescue mobilization and standard G-CSF mobilization using multiparameter flow cytometry. HSPCs were identified by size and granularity (forward and side scatter) and CD34 expression (as exemplified in Fig. 1A). Representative fluorescence histograms of each receptor and the MFIRs are depicted on the left side of Figs. 1B through 1E. CXCR4 was weakly expressed on CD34+ cells of both treatment groups as shown by median MFIRs of 1.8 (G-CSF) and 2.0 (G-CSF plus plerixafor, Fig. 1B, middle) and median percentages of CXCR4-positive CD34+ cells of 4.3 (G-CSF) and 14.2 (G-CSF plus plerixafor, Fig. 1B, right). As CXCR4 levels were directly determined in fresh leukapheresis products the low levels were not caused from down regulation during density centrifugation or freezing and thawing. In contrast, the expression of VLA-4, as determined by flow cytometric analysis of CD49d (α-subunit of VLA-4), was high on the entire CD34+ population throughout all patients, independent of the mobilizing regimen (Fig. 1C). Similarly, LFA-1, as determined by analysis of CD11a (α-subunit of LFA-1) levels (Fig. 1D), and CD44 (Fig. 1E) were highly expressed on mobilized CD34+ cells of all patients and did not differ between G-CSF and G-CSF plus plerixafor–mobilized CD34+ cells.

Collectively, our data show that CD34+ cells collected after G-CSF plus plerixafor–induced rescue mobilization or standard G-CSF administration exhibit similar CXCR4 and adhesion receptor expression profiles despite different mechanisms of release from the BM.

### The addition of plerixafor to G-CSF causes increased mobilization of primitive CD133+/CD34+/CD38– HCs

To compare the HC subsets collected after either mobilization regimen, we characterized CD133, CD34, and CD38 expression in leukapheresis products via flow cytometry. Although CD34 is widely used as HSPC marker,^46^,^47^ CD34–/CD38–/Lin– HCs are capable of repopulating immunodeficient mice.^48^ Therefore, we additionally analyzed the alternative HSPC marker CD133,^49^ which is expressed on primitive CD34–/CD38–/Lin–50 as well as on CD34+ HCs, where it marks a subpopulation enriched for repopulating cells.^51^,^52^ Within the CD34+ population, CD38– cells represent a subset with extensive proliferation and repopulating capacity,^53^,^54^ while CD38+ cells express differentiation markers and have limited repopulating capacities.^55^

**Figure 2:**
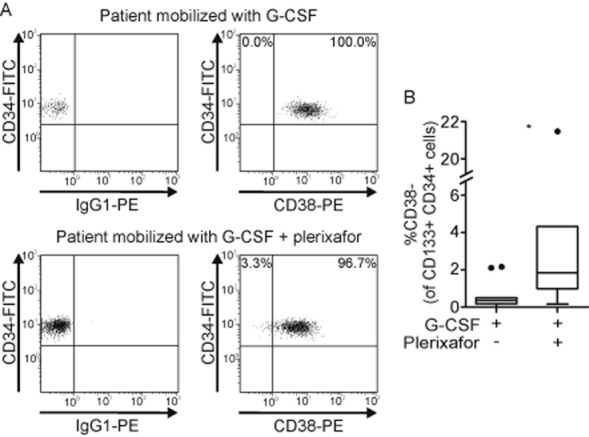
CD34 and CD38 expression on CD133+ HCs in leukapheresis products. (A) The dot plots show the surface expression of CD34 and CD38 on pregated CD133+ HCs and are representative for 11 leukapheresis products collected after G-CSF (top) and seven leukapheresis products collected after G-CSF plus plerixafor administration (bottom). (B) Percentages of CD38– cells within the CD133+/CD34+ cell population in G-CSF (n = 11) compared to G-CSF plus plerixafor (n = 7). *p < 0.05.

Figure 2A illustrates a typical CD34 and CD38 expression profile of CD133+ HCs in leukapheresis products collected after G-CSF (top) and G-CSF plus plerixafor administration (bottom). In all patients the main population of CD133+/CD34+ HCs also expressed CD38 (exemplified in Fig. 2A). While leukapheresis products collected after G-CSF treatment contained no or very small percentages of CD133+/CD34+/CD38– HCs, G-CSF plus plerixafor induced the collection of a significantly larger subpopulation of early CD133+/CD34+/CD38– HCs (median, 0.39% compared to 1.84%; p = 0.037, Fig. 2B).

### Plerixafor rescues HSPC mobilization in poor mobilizers

**Figure 3:**
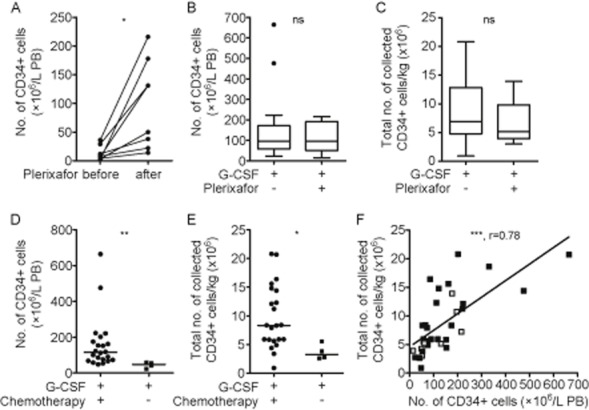
Plerixafor rescues HSPC mobilization in poor mobilizers. (A) Numbers of preapheresis CD34+ cells (×10^6^/L PB) 1 day before and the next morning after plerixafor injection in poor mobilizers as determined by flow cytometry (n = 8). (B) Comparison of preapheresis CD34+ cell counts (×10^6^/L PB) in good mobilizers receiving G-CSF (n = 28) and poor mobilizers receiving G-CSF plus plerixafor (n = 8). (C) Numbers of CD34+ cells/kg collected after G-CSF– (n = 26) or G-CSF plus plerixafor–induced mobilization (n = 8). Results are illustrated in box plot format, indicating the median. The 25th and 75th percentiles are marked by the edges of the box and the minimum and maximum observations (excluding outliers) are marked by the whiskers. Outliers are defined as cases that are more than 1.5-fold the interquartile range away from the edges of the box and are indicated as dots. (D) Preapheresis CD34+ cell numbers (×10^6^/L PB) of good mobilizers who did (n = 22) or did not (n = 4) receive chemotherapy immediately before G-CSF. (E) Total CD34+ cell numbers/kg collected from good mobilizers, who received chemotherapy before G-CSF (n = 22) or not (n = 4). (F) Correlation of preapheresis CD34+ cell numbers (×10^6^/L PB) with total CD34+ cells/kg collected via leukapheresis. (■) Patients treated with G-CSF (n = 27); (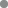) patients treated with G-CSF plus plerixafor (n = 8). *p < 0.05; **p < 0.01; ***p < 0.001; ns = not significant.

To determine how the addition of plerixafor to G-CSF in poor mobilizers affects mobilization efficiency, we first compared circulating CD34+ cell counts 1 day before and the next morning after plerixafor administration before leukapheresis. Figure 3A shows that plerixafor induced a 12-fold elevation of the median number of circulating CD34+ cells (×10^6^/L PB) from 7.5 to 90.5. Further, we found that G-CSF plus plerixafor induced the mobilization of similar numbers of CD34+ cells (×10^6^/L) to the PB (median, 96.5 × 10^6^ cells/L) like G-CSF alone in good responders (median, 95.0 × 10^6^ cells/L; Fig. 3B), as determined shortly before leukapheresis, and resulted in similar yields of CD34+ cells/kg by leukapheresis (median, 5.2 × 10^6^ and 6.9 × 10^6^ cells/kg; Fig. 3C). Of note, within G-CSF–treated patients, those who underwent chemotherapy before G-CSF treatment had significantly higher CD34+ cell counts (×10^6^/L PB; median, 115.0 × 10^6^ compared to 48.0 × 10^6^ cells/L; Fig. 3D) and CD34+ cell yields/kg (median, 8.3 × 10^6^ compared to 3.3 × 10^6^ cells/kg; Fig. 3E). Preapheresis CD34+ cell numbers (×10^6^/L) in the PB of the whole patient collective correlated with the number of CD34+ cells/kg yielded by subsequent leukapheresis (Fig. 3F), which confirms peripheral CD34+ counts to be helpful indicators to predict mobilization success.

Together, our data show that the on-demand use of plerixafor potently induces mobilization of CD34+ cells in patients poorly responding to G-CSF (with or without chemotherapy) and facilitates mobilization rates similar to those of good mobilizers.

### CXCR4, VLA-4, LFA-1, and CD44 expression on mobilized CD34+ cells is independent of mobilization efficiency

**Figure 4:**
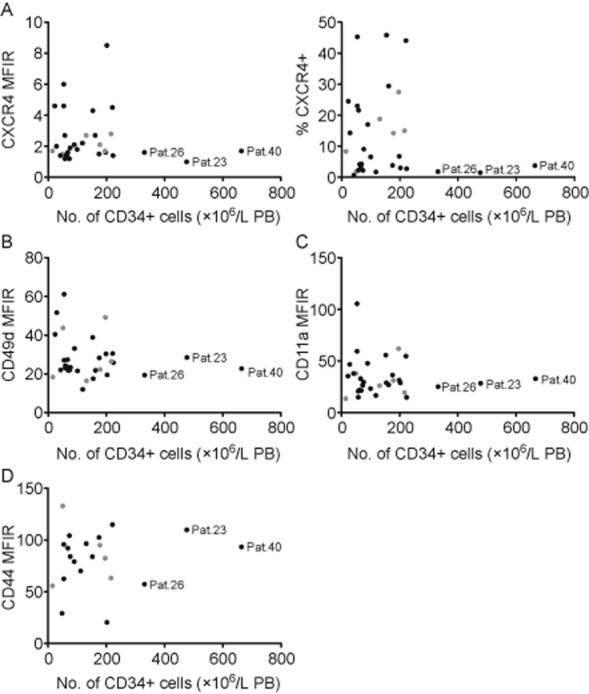
CXCR4, CD49d, CD11a, and CD44 expression on mobilized CD34+ cells are independent of mobilization efficiency. Preapheresis CD34+ cell numbers (×10^6^/L PB) were correlated with MFIRs of CXCR4 (A, left, n = 31), percentages of CXCR4+ cells within the CD34+ population (A, right, n = 31) and MFIRs of CD49d (B, n = 32), CD11a (C, n = 32), and CD44 (D, n = 21) on CD34+ cells. (●) Patients receiving G-CSF; (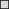) patients receiving G-CSF plus plerixafor.

Next, we evaluated whether the surface expression of receptors involved in HSPC retention in the BM is related to mobilization efficiency. The relations of preapheresis CD34+ cell numbers (×10^6^/L PB) with expression of respective receptors are shown in Fig. 4 and illustrate that the surface levels (MFIRs) of CXCR4 (Fig. 4A, left) and percentages of CXCR4 expressing CD34+ cells (Fig. 4A, right) and the surface levels of CD49d (VLA-4, Fig. 4B), CD11a (LFA-1, Fig. 4C), and CD44 (Fig. 4D) do not correlate with mobilization efficiency as analyzed in the total patient cohort. The separate analysis of both treatment groups similarly did not show any correlation (data not shown). However, it is worth mentioning that three patients displaying particularly high mobilization response (>300 × 10^6^ CD34+ cells/L PB) exhibit a low expression of CXCR4, CD49d, and CD11a.

### Transplantation with HCs mobilized via G-CSF or G-CSF plus plerixafor results in similar time to engraftment

**Figure 5:**
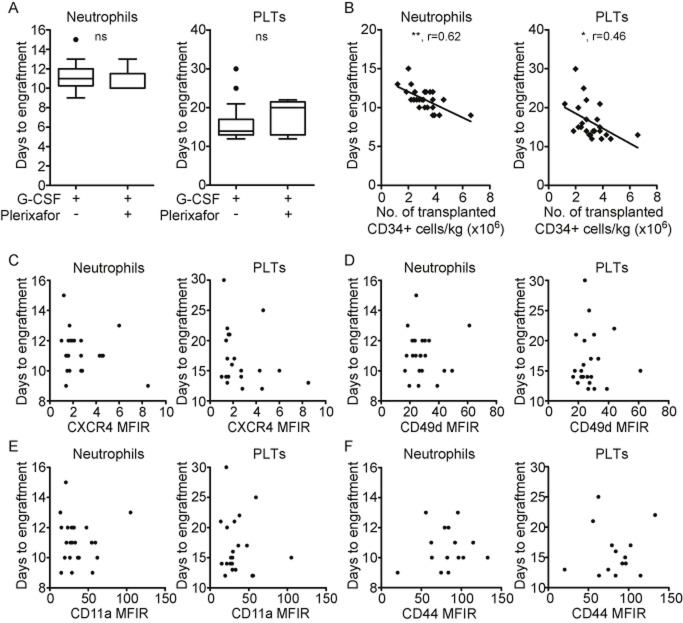
Transplantation with HCs mobilized via G-CSF or G-CSF plus plerixafor results in similar time to engraftment. (A) The days to neutrophil (left, n_(G)_ = 20, n_(G+P)_ = 6) and PLT engraftment (right, n_(G)_ = 19, n_(G+P)_ = 5) after transplantation with G-CSF– or G-CSF plus plerixafor–mobilized HCs are shown in box plot format. (B) Correlation of transplanted CD34+ cell numbers/kg (mobilized by G-CSF or G-CSF plus plerixafor) with time to neutrophil (left, n = 26) and PLT engraftment (right, n = 24). Correlations of neutrophil (left) and PLT engraftment (right) with surface levels of CXCR4 (C, n_(Neutrophils)_ = 23 and n_(PLTs)_ = 21), CD49d (D, n_(Neutrophils)_ = 24 and n_(PLTs)_ = 22), CD11a (E, n_(Neutrophils)_ = 24 and n_(PLTs)_ = 22), and CD44 (F, n_(Neutrophils)_ = 15 and n_(PLTs)_ = 14). G = G-CSF; G+P = G-CSF plus plerixafor. *p < 0.05; **p < 0.01; ns = not significant.

A rapid engraftment of transplanted HCs in patients after high-dose chemotherapy is essential in reconstituting the immune system and minimize the risk of severe infections. Therefore, we compared the time to of hematopoietic recovery (as defined by time to neutrophil and PLT engraftment) in 26 patients transplanted with autologous HCs collected after either mobilization regimen. Neutrophil engraftment after transplantation with HCs mobilized by G-CSF resulted in neutrophil engraftment after a median of 11 days, comparable to that of neutrophil engraftment after G-CSF plus plerixafor–induced mobilization (median, 10 days; Fig. 5A, left). PLT engraftment occurred after a median of 14 days in patients receiving G-CSF–mobilized HCs and after a median of 20 days in patients receiving G-CSF plus plerixafor-mobilized HCs (Fig. 5A, right). Next, we investigated which variables influenced time to engraftment. As reported previously for G-CSF–mobilized HCs, in our cohort of G-CSF and G-CSF plus plerixafor–treated patients, the number of transplanted CD34+ cells/kg inversely correlated with time to neutrophil engraftment (Fig. 5B, left) and weakly inversely correlated with time to PLT engraftment (Fig. 5B, right). To further assess the influence of CXCR4 and adhesion molecule expression on CD34+ cells in apheresis products on engraftment we related expression levels of each receptor to neutrophil and PLT engraftment time. As shown in Figs. 5C through 5F, none of the CXCR4, CD49d, CD11a, or CD44 surface expression levels were related to neutrophil or PLT engraftment. Our results show that G-CSF and G-CSF plus plerixafor mobilize HSPCs with similar engraftment kinetics and that the time to engraftment primarily depends on the number of transplanted CD34+ HCs but not on CXCR4 and adhesion molecule expression.

## Discussion

Limited data are currently available comparing the phenotype and engraftment properties of HSPCs collected after G-CSF plus plerixafor–induced rescue mobilization compared to traditional G-CSF administration. In our exploratory study on a total of 37 patients we found that while the on-demand addition of plerixafor to G-CSF in poor mobilizers altered the subset composition in leukapheresis products in favor of more primitive CD133+/CD34+/CD38– HCs, the CXCR4 and adhesion molecule expression profiles were similar on the bulk CD34+ population collected after both mobilization regimens. In line, plerixafor-induced rescue mobilization was similarly effective in mobilizing CD34+ cells and caused equal neutrophil and PLT engraftment after transplantation like G-CSF in good mobilizers.

Previous studies suggested that a modulation of adhesion receptor expression on BM HSPCs occurs during G-CSF administration, which might be functionally involved in the mobilization process. Specifically, CXCR4 surface expression was found to be dynamically modulated in response to G-CSF,^25^ and CXCR4, VLA-4, and LFA-1 surface levels were suggested to be down regulated during mobilization into the circulation.^25^,^56^,^57^ Although plerixafor is thought to elicit less pronounced effects on the BM microenvironment than G-CSF, HSPCs mobilization to the PB might be accompanied by alterations in their adhesion molecule expression. For example, plerixafor has recently been shown to induce release of CXCL12 from BM stromal cells into the PB,^58^ which in turn might affect CXCR4 expression on HSPCs, for example, by induction of CXCR4 internalization. Studies in an autologous transplantation rhesus macaque model demonstrated that plerixafor-mobilized CD34+ cells have higher VLA-4 and CXCR4 levels as G-CSF–mobilized CD34+ cells.^59^ In contrast, we did not detect any differences in the adhesion molecule expression on mobilized CD34+ cells, whether plerixafor was used or not. Importantly, our patients received G-CSF until the day before plerixafor injection and therefore might exhibit G-CSF–induced alterations on mobilized CD34+ cells. These receptor modulations alone might have not been sufficient to initiate pronounced mobilization but might facilitate release from the BM in addition to plerixafor-specific mechanisms. Our interpatient comparison of G-CSF plus plerixafor– versus G-CSF–mobilized subsets illustrated that the addition of plerixafor also induced the release of more primitive CD133+/CD34+/CD38– cells in poor mobilizers compared to G-CSF alone in good mobilizers. A previous study showed that the supplementation of plerixafor in a G-CSF–based regimen increased the percentage of CD38– cells in the circulating CD133+/CD34+ population within individual MM and NHL patients^60^ and another study reported elevated percentages of CD133+/CD38– within CD34+ cells if plerixafor was added to G-CSF instead of cyclophosphamide.^61^ Together these data show that the different mobilizing mechanisms of plerixafor and G-CSF cause the collection of different HC subset combinations.

A rapid hematopoietic recovery after HC transplantation is essential to reduce the risk of secondary infections. Recently, a minimal delay in engraftment independent of the CD34+ cell dose was associated with mobilization by G-CSF plus plerixafor in comparison to G-CSF.^62^ Our data (albeit determined in a small cohort) do not support this suggestion, as we have observed that transplantation with HCs mobilized by either regimen result in similar engraftment times. Although CD34+/CD38– cells possess extensive proliferation and repopulation potentials,^53^,^54^ greater percentages within CD34+ cells or total transplanted numbers were not related to faster engraftment (data not shown). However, effects on long-term hematopoiesis need to be further investigated. Furthermore, engraftment time after transplantation did not correlate with the expression of BM homing and retention receptors on CD34+ cells (that one could expect) but was strictly correlated with the number of transplanted CD34+ cells, supporting a previous study^63^ and underlining its dominant role in determining engraftment. A recent report shows that patients with prolonged time to neutrophil engraftment (>19 days) exhibited lower VLA-4 levels on collected CD34+ cells.^64^ Importantly, all our patients expressed high VLA-4 levels on collected CD34+ cells, which might exceed a certain threshold necessary to effectively mediate HSPC homing and retention within the BM. In line, neutrophil engraftment occurred faster in our entire patient cohort (≤17 days), which could further explain the lack of correlation. Although CXCR4 is essential for homing into the BM, we did not observe any association of CXCR4 levels and engraftment. Interestingly, previous studies have shown that CXCR4 expression on CD34+ cells is dynamically regulated and that CD34+ cells can release CXCR4 from intracellular stores to the cell surface upon cytokine stimulation.^65^ Therefore, we suggest that the low CXCR4 expression on collected CD34+ cells might be significantly increased upon injection into the recipient to facilitate migration into the BM.

Collectively, our results suggest that the use of plerixafor on-demand releases HSPCs that are qualitatively equivalent to HSPCs mobilized by G-CSF, in terms of BM homing and retention receptor expression and engraftment potential. Therefore, our study, albeit using a limited patient number, supports the use of plerixafor for poor mobilizers to rescue HSPC mobilization and facilitate their treatment with potentially curative high-dose therapy and HC transplantation.

## Abbreviations

BM: marrow
HC(s): hematopoietic cell(s)
HSPC(s): hematopoietic stem and progenitor cell(s)
MFIR(s): mean fluorescence intensity value(s) in relation to isotype control
MM: multiple myeloma
NHL: non-Hodgkin's lymphoma
PB: peripheral blood

## Supporting Information

Table S1 Detailed patient characteristics
