# Supplementary material for: The CXCR4 and adhesion molecule expression of CD34+ hematopoietic cells mobilized by “on-demand” addition of plerixafor to granulocyte–colony-stimulating factor
Source: Transfusion. 2014 Mar 28;54(9):2325–35. doi: 10.1111/trf.12632 (PMC4215600; doi:10.1111/trf.12632)
Supplement: Table S1 — Detailed patient characteristics [file trf0054-2325-sd1.docx]

SupplementalTable 1: Detailed patient characteristics. R-DHAP, rituximab - dexamethasone, cytarabine and cisplatin; DHAP, dexamethasone, cytarabine and cisplatin; BEACOPP, bleomycin, etoposide, doxorubicin, cyclophosphamide, vincristine, procarbazine and prednisone; CHOEP, cyclophosphamide, doxorubicin, vincristine and prednisone plus etoposide.

| Patient | Sex | Age | Diagnosis | Chemotherapy regimen | Time of plerixafor injection (days after first G-CSF injection) |
| --- | --- | --- | --- | --- | --- |
| Patient 6 | m | 69 | Non-Hodgkin´s lymphoma | Cyclophosphamide | not required |
| Patient 7 | m | 69 | Non-Hodgkin´s lymphoma | R-DHAP | not required |
| Patient 10 | f | 62 | Non-Hodgkin´s lymphoma | - | 4 |
| Patient 14 | m | 34 | Hodgkin´s lymphoma | BEACOPP | not required |
| Patient 15 | m | 18 | Acute lymphocytic leukemia | Cyclophosphamide | not required |
| Patient 16 | m | 68 | Multiple myeloma | Cyclophosphamide | not required |
| Patient 17* | m | 43 | Multiple myeloma | Cyclophosphamide | not required |
| Patient 19 | m | 69 | Multiple myeloma | Cyclophosphamide | not required |
| Patient 20 | m | 68 | Multiple myeloma | - | not required |
| Patient 21 | m | 72 | Non-Hodgkin´s lymphoma | R-DHAP | 10 |
| Patient 22 | f | 35 | Non-Hodgkin´s lymphoma | - | not required |
| Patient 23 | m | 56 | Multiple myeloma | Cyclophosphamide | not required |
| Patient 24 | f | 45 | Hodgkin´s lymphoma | DHAP | not required |
| Patient 25 | m | 38 | Non-Hodgkin´s lymphoma | R-DHAP | not required |
| Patient 26 | m | 52 | Non-Hodgkin´s lymphoma | DHAP | not required |
| Patient 27 | m | 26 | Non-Hodgkin´s lymphoma | CHOEP | not required |
| Patient 28 | m | 61 | Multiple myeloma | Cyclophosphamide | not required |
| Patient 29 | f | 49 | Non-Hodgkin´s lymphoma | DHAP | not required |
| Patient 30 | m | 73 | Multiple myeloma | - | not required |
| Patient 31 | f | 46 | Non-Hodgkin´s lymphoma | - | not required |
| Patient 32 | f | 44 | Acute myeloid leukemia | - | not required |
| Patient 33 | f | 54 | Multiple myeloma | Cyclophosphamide | not required |
| Patient 34 | f | 65 | Non-Hodgkin´s lymphoma | R-DHAP | not required |
| Patient 35 | f | 68 | Multiple myeloma | - | 5 |
| Patient 36* | f | 62 | Multiple myeloma | - | 4 |
| Patient 37* | f | 61 | Multiple myeloma | - | 4 |
| Patient 38 | f | 68 | Scleromyxedema | Cyclophosphamide | 5 |
| Patient 39 | m | 47 | Multiple myeloma | Cyclophosphamide | not required |
| Patient 40 | m | 38 | Non-Hodgkin´s lymphoma | Cyclophosphamide | not required |
| Patient 41 | f | 70 | Multiple myeloma | Cyclophosphamide | not required |
| Patient 42 | f | 69 | Multiple myeloma | Cyclophosphamide | not required |
| Patient 43 | m | 62 | Multiple myeloma | Cyclophosphamide | not required |
| Patient 45 | f | 30 | Non-Hodgkin´s lymphoma | Cyclophosphamide | 7 |
| Patient 46 | m | 71 | Multiple myeloma | Cyclophosphamide | not required |
| Patient 47 | m | 68 | Multiple myeloma | Cyclophosphamide | not required |
| Patient 48 | f | 35 | Non-Hodgkin´s lymphoma | Cyclophosphamide | 7 |
| Patient 49 | m | 36 | Hodgkin´s lymphoma | DHAP | not required |

* The asterisks marks patients where platelet engraftment was defined as time point where platelet count remained stable without any need of transfusions (count below the regular 50,000)
